# Using concept mapping to inform the development of a transitional reintegration intervention program for formerly incarcerated people with HIV

**DOI:** 10.1186/s12913-019-4595-y

**Published:** 2019-10-28

**Authors:** Tony Antoniou, Sharmistha Mishra, Flora Matheson, Diane Smith-Merrill, Laurel Challacombe, Janet Rowe, Anne Marie DiCenso, Fiona G. Kouyoumdjian, Wendy Wobeser, Claire Kendall, Mona Loutfy, Jenkin Tsang, Lauren Kanee, Carol Strike

**Affiliations:** 1 Department of Family and Community Medicine, St. Michael’s Hospital and University of Toronto, 410 Sherbourne Street, 4th Floor, Toronto, Ontario M4X 1K2 Canada; 2 Li Ka Shing Knowledge Institute, St. Michael’s Hospital, Toronto, Ontario Canada; 3 Centre for Urban Health Solutions, St. Michael’s Hospital, Toronto, Ontario Canada; 4 Department of Medicine, University of Toronto, Toronto, Ontario Canada; 5 Dalla Lana School of Public Health, University of Toronto, Toronto, Ontario Canada; 6 Centre of Criminology and Sociolegal Studies, University of Toronto, Toronto, Ontario Canada; 7 HIV/AIDS Regional Services, Kingston, Ontario Canada; 8 Canadian AIDS Treatment Information Exchange, Toronto, Ontario Canada; 9 Prisoners HIV/AIDS Support Action Network, Toronto, Ontario Canada; 10 Parkdale Queen West Community Health Centre, Toronto, Ontario Canada; 11 Department of Family Medicine, McMaster University, Hamilton, Ontario Canada; 12 Queen’s University, Kingston, Ontario Canada; 13 Department of Family Medicine, University of Ottawa, Ottawa, Ontario Canada; 14 C.T. Lamont Primary Health Care Research Centre, Bruyère Research Institute, Ottawa, Ontario Canada; 15 Women’s College Research Institute, Women’s College Hospital, Toronto, Canada

**Keywords:** HIV, Health care access, Incarceration, Concept mapping

## Abstract

**Background:**

Accessing HIV-related care is challenging for formerly incarcerated people with HIV. Interventions informed by the perspectives of these individuals could facilitate engagement with care and address competing priorities that may act as barriers to this process.

**Methods:**

We used concept mapping to identify and prioritize the main obstacles to engaging with HIV-related care following prison release. In brainstorming sessions, formerly incarcerated people with HIV generated responses to a focused prompt regarding the main barriers to reengaging with care. These were consolidated in 35 statements. Next, participants sorted the consolidated list of responses into groups and rated each from lowest to highest in terms of its importance and feasibility of being addressed. We used cluster analysis to generate concept maps that were interpreted with participants.

**Results:**

Overall, 39 participants participated in brainstorming sessions, among whom 18 returned for rating and sorting. Following analysis, a seven-cluster map was generated, with participants rating the ‘Practical Considerations’ (e.g. lack of transportation from prison) and ‘Survival Needs’ (e.g. securing housing and food) clusters as most important. Although ratings were generally similar between women and men, women assigned greater importance to barriers related to reconnecting with children.

**Conclusions:**

Using concept mapping, we worked with formerly incarcerated people with HIV to identify and prioritize key challenges related to accessing health and social services following prison release. Transitional intervention programs should include programs and processes that address meeting basic subsistence needs and overcoming logistical barriers related to community re-entry.

## Background

The transition from prison to community is challenging for formerly incarcerated persons with HIV [1]. Yet programs to facilitate re-engagement with HIV care at the time of release are often not available [1, 2]. Consequently, formerly incarcerated individuals with HIV are at risk of negative health outcomes related to interruptions in antiretroviral therapy and continuity of care [3–7]. Even when antiretroviral prescriptions and medical appointments are provided at the time of release, several studies suggest that linkage to and retention in care remain suboptimal. In one study, only 5% of persons with HIV who were prescribed antiretroviral therapy at the time of release filled their prescriptions within the ensuing 10 days, and only 28% linked with an HIV clinic within 1 month [8]. Similar results were observed in another study evaluating post-release interventions in ten cities in the United States, with only one-third of 867 prisoners living with HIV released to the community being retained in HIV care 6 months following release [9]. Successful post-release linkage to care appears to be less likely among formerly incarcerated women with HIV relative to men [10]. Specifically, in one study, only 38% of women had accessed care within 30 days of release, compared with 60% of men [11]. In another study, women were significantly less likely to have a usual care provider at 6 months following prison release and less likely to be virologically suppressed at this time point (18% versus 30%) [12]. Although reasons for these differences are unclear, formerly incarcerated women with HIV are three-times more likely to experience intimate partner violence than men, a finding which has been endorsed by women as being directly related to risk of recidivism, substance use relapse and disrupted health care continuity [13].

Inadequate post-release linkage to HIV care also has repercussions for the health care system, with hospital emergency departments becoming important venues of care for formerly incarcerated people with HIV [14]. In addition to providing medical care, emergency departments are visited by these individuals for reasons related to the social and psychiatric instability associated with community reentry, including depression, homelessness and substance use. Importantly, a significant proportion (23%) of emergency visits resulted in hospitalization, further highlighting the burden imparted to the healthcare system by inadequate post-release linkage to HIV health and social support services [14].

Because inadequate linkage to health and social services is associated with negative consequences for both individuals and the health care system, interventions that help formerly incarcerated people with HIV successfully bridge the transition from prison to community are required. A necessary prerequisite for developing such interventions is understanding the barriers formerly incarcerated people with HIV must overcome to access HIV care. Several qualitative studies have demonstrated that the need to address competing non-medical issues at the time of release, such as unstable housing, reinstating health insurance and treatment for co-existing mental health illness and substance use, may be prioritized over medical care by formerly incarcerated people with HIV [15–19]. However, use of these studies for informing the implementation of programs may be limited for several reasons. First, in order to inform local program development, findings generated from qualitative research must be transferable to varied contexts [20]. Although selected aspects of earlier research would be contextually transferable to our setting, certain barriers to engagement with care would not be applicable in Canada. For example, Canada has a system of publicly funded health insurance that covers physician visits. Consequently, in contrast to jurisdictions in which many earlier studies were conducted, re-instating health insurance would not be a barrier to engaging with care for formerly incarcerated individuals with HIV in Canada. However, this is not true of prescription drug coverage, where coverage and eligibility criteria vary among Canada’s provinces. Second, participants are typically not involved in the analysis of data generated in qualitative research, thereby leaving the bulk of interpretation and theorizing to the research team rather than the individuals who would be using any resultant interventions.

To inform the design of a context-specific program in Ontario, Canada and ensure that participants have a voice in the research process and development of an intervention, we used a participatory approach called concept-mapping to study barriers preventing formerly incarcerated people with HIV from accessing medical care and social services at the time of release [21, 22]. In light of sex-based differences in post-release outcomes, we were also interested in contrasting the perspectives of women and men on the most salient challenges and priorities in the period immediately following release. We also sought to apply these findings towards the creation of a transitional intervention program for formerly incarcerated people with HIV.

## Methods

### Study design

We used concept mapping to solicit, structure and prioritize the perspectives of formerly incarcerated people with HIV in Ontario, Canada. Concept mapping is a structured, participatory approach that involves participants at all stages of the research process, including data analysis and interpretation, and has been extensively used in program planning and evaluation [21, 22]. This methodology integrates group process activities on a topic of interest with quantitative methods to produce a visual representation of the relationship between participant ideas. This study was approved by the Research Ethics Board of the University of Toronto, Toronto, Canada.

### Participants

We worked with six community-based agencies and HIV clinics from across Ontario to purposively sample formerly incarcerated people with HIV who were at least 18 years of age and who could speak to the experience of attempting to re-engage with health and social services following prison release. At each site, a staff member identified prospective participants as clients of the agency who met the inclusion criteria and provided information about the study. A total of 47 individuals from the 6 sites (ranging from 4 individuals to 13 individuals per site) were invited to participate in brainstorming sessions that were held between September 2015 and August 2016, eight of whom did not ultimately arrive for the session. Participants recruited for the brainstorming sessions were informed of the subsequent sorting and rating activities and invited to participate in those sessions, held between May and September 2017.. We conducted all study activities in the offices of community-based agencies and clinics from which participants were recruited. For participants who were uncomfortable completing study activities in a group setting, individual meetings with a member of the study team were arranged. Written informed consent was obtained from all individual participants included in the study. The consent form included a separate question seeking consent for the research team to contact study participants for subsequent sorting and rating activities. Because of the potentially sensitive nature of the study, we verbally reinforced the importance of respecting the privacy of co-participants outside of the study setting.

### Brainstorming

We developed a brainstorming prompt statement in collaboration with representatives of agencies involved in the care of formerly incarcerated people with HIV and community members. At each brainstorming session, participants were asked to individually complete a brief sociodemographic questionnaire and through group interaction, generate statements in response to the prompt statement “Why do people with HIV have a hard time connecting with health and support services after release from prison?” To help participants understand the prompt, we provided several illustrative examples, such as ‘stop taking their HIV medication’, ‘don’t go for appointments with a family doctor’ and ‘can’t be found by providers or case workers’. Participants’ responses were recorded on flip-charts and then entered verbatim into a database for subsequent statement consolidation. Overall, 39 participants (seven group sessions, two individual sessions) generated 96 statements.

### Statement consolidation

We used a process recommended by Kane and Trochim to consolidate the generated statements into a shorter list that would be manageable for sorting and rating by participants [22]. Specifically, we assigned key words to each statement, sorted the statements based on these terms and removed statements representing duplicate ideas and/or those that did not address the prompt. We then combined specific statements describing the same or overlapping ideas and edited the consolidated statements for common wording and simplicity. For example, the statements “nobody to pick me up from prison”, “no ride from jail” and “no way to get home after getting out” were combined into ‘no transportation when released’. This process resulted in a list of 35 consolidated statements.

### Sorting and rating

The sorting and rating activity was completed by 18 participants who had consented to be contacted again by the research team following the brainstorming activity. Each participant was provided with a deck of 35 cards corresponding to the 35 consolidated statements, with one statement per card. Participants were instructed to organize statements into groups that made sense to them and provide each group with a name that reflected the theme of the summarized ideas. Groupings could occur in any manner, with no limits on the number of groups that could be generated, with the following restrictions: statements could not all be placed in the same pile, each statement could not form a pile on its own, piles could not be organized by priority or other criteria (e.g. true and false, does not apply to me) and dissimilar statements could not be grouped together into piles labelled ‘other’ or ‘miscellaneous’. Next, participants were instructed to rate all statements on two dimensions: importance as a barrier to linking with post-release and feasibility of addressing barrier within the next 3 to 5 years. Rating was conducted using a 5-point Likert scale for each dimension, with ‘1’ indicating ‘unimportant’ and not at all feasible’, and ‘5’ indicating ‘extremely important’ and ‘extremely feasible’. The sample size for each concept mapping activity was sufficient for attaining valid and reliable results (i.e. > 20 participants for brainstorming, > 10 participants for rating and sorting) [23, 24].

### Statistical analysis

We used Concept Systems Software version 4.0 (Concept System Program, Concept Mapping Incorporated, Ithaca, New York) for all analyses. The analysis proceeded in several steps. First, each participant’s sorting data were used to generate a similarity matrix displaying the number of participants who sorted each pair of statements together. Next, multidimensional scaling analysis was used to assign each statement a two dimensional coordinate (x, y) and position statements in relation to one another based on how frequently they were paired during the sorting activity [22, 25]. The resultant point map provides a visual representation of the relative similarity of statements based on their proximity to each other, such that points on the map that are closer together represent statements that are more frequently sorted with one another in the sorting task, while points that are further apart represent statements that are sorted together less frequently [25]. Last, hierarchical cluster analysis partitioned the point map into non-overlapping clusters representing groups of statements that are conceptually or thematically similar [22]. A stress value between zero and one was calculated to indicate the goodness of fit of statement configurations, where a smaller value is indicative of a smaller discrepancy between maps and the input similarity matrix. Values between 0.205 and 0.365 represent a good fit for field-based projects [25]. In addition, bridging values were generated for each statement. Ranging from zero to one, bridging values reflect how frequently statements were sorted together, with low values indicative of statements sorted together primarily with statements that are close to it on the map [22]. Conversely, higher values indicate that a statement ‘bridges’ two or more clusters. For example, a statement with a bridging value of 1 indicates that this statement could be potentially sorted with every cluster. The bridging values of each statement within a cluster were then averaged to produce a bridging value for the cluster, with low values demonstrating that statements within a particular cluster were commonly sorted together. Finally, we generated ‘go-zone’ matrices and Pattern Match charts. Go-zone matrices assign *x*- and *y*-axes to importance and feasibility ratings by participants where each statement is assigned coordinates based on its respective mean rating. Lines corresponding to the mean rating for each axis divide the graph into four quadrants (low-low, low-high, high-low, and high-high). Items in the top right quadrant were those that are rated as highly important and feasible, whereas those in the bottom left quadrant were rated as less important and feasible to address. Consequently, the high-high quadrant is considered the ‘go-zone’ as it contains those ideas rated most highly on both criteria. Pattern matches are ladder graphs that display a comparison of average cluster ratings between two variables or groups. For our study, we compared cluster importance and feasibility between women and men.

Participants were engaged in the interpretation of data during the rating and sorting exercises. Specifically, data entry occurred during the sessions and participants were asked to reflect upon which cluster solution (i.e. the total number of clusters) best reflected their ideas and whether the grouping of specific groups of statements into distinct clusters made sense to them Individual sorting and rating exercises were not repeated as part of this process. Furthermore, participants were asked to discuss the nature of services that would be required to facilitate re-engagement with care.

## Results

### Participants

The mean age of the participants was 50 years and 16 (41%) were women (Table 1). The majority of participants did not receive antiretroviral prescriptions or referrals for health and social services at the time of release.

**Table 1:** Characteristics of Study Participants

Characteristic	*n* = 39
Age [years] (Mean, SD)	50.0 (8.8)
Sex	
Male	23 (59.0%)
Female	16 (41.0%)
Region of Ontario	
Toronto	19 (48.7%)
Ottawa/Kingston	12 (32.8%)
Northern Ontario	8 (20.5%)
Current status in Canada	
Canadian citizen	36 (92.3%)
Landed immigrant/permanent resident	1 (2.6%)
Not available	2 (5.1%)
Currently receiving HIV treatment	35 (89.7%)
Annual Income $40,000 or less	38 (97.4%)
Highest level of Education Completed	
Less than grade 9	5 (12.8%)
Some high school	10 (25.6%)
Completed high school	9 (23.1%)
Trade or technical training	3 (7.7%)
Post-secondary	12 (30.8%)
Risk factor for HIV acquisition^a^	
Sex	22 (56.4%)
Injection drug use	20 (51.3%)
Don’t know	2 (5.1%)
Post-release planning	
Referral to family doctor or HIV specialist	13 (33.3%)
Prescription for HIV medication provided	13 (33.3)
Referral for social services	4 (10.3%)
Referral to community-based AIDS Service Organization	6 (15.4%)

^a^Participants may have selected more than one option

### Cluster map

**Fig. 1:**
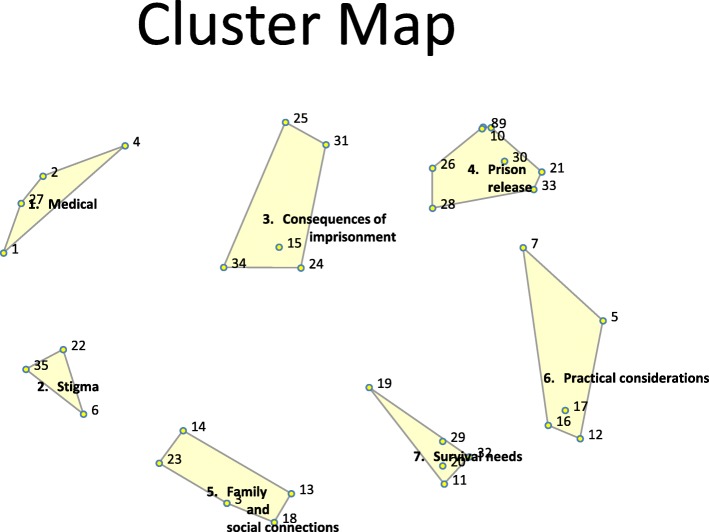
Seven cluster concept map

Based on participants’ sorting of statements and interpretation, a final cluster map comprising seven dimensions was generated (Fig. 1). The specific clusters included Medical (4 statements), Stigma (3 statements), Consequences of Imprisonment (5 statements), Prison Release (8 statements), Practical considerations (5 statements), Family and Social Connections (5 statements) and Survival Needs (5 statements). The concept map had a stress value of 0.23, indicating a good fit between the sorting data and cluster configurations. The cluster bridging values ranged from 0.18 to 0.87. The ‘Medical’ cluster, with a high bridging value of 0.87, consists of statements that were also commonly sorted with statements in other clusters. In contrast, the ‘Prison Release’ cluster, with a bridging index of 0.18, includes statements that were grouped together most frequently by participants and are therefore firmly ‘anchored’ to their position on the map.

### Rating

Overall, participants tended to assign higher ratings for importance than feasibility to each cluster (Table 2). The clusters rated most important by participants were ‘Practical Considerations (mean 4.4), reflecting logistical matters related to community re-entry such as lack of transportation (mean 4.2) and re-instating drug coverage (mean 4.7), and ‘Survival Needs’ (mean 4.6), a domain encompassing statements such as ‘safety and protection from abuse (mean 4.5), securing housing (mean 4.8) and ‘need to secure food’ (mean 4.7). Despite being assigned a high mean rating on importance, the ‘Survival Needs’ cluster was among the lowest rated in terms of feasibility (mean 3.3). Other domains with relatively low mean ratings for feasibility included ‘Prison Release’ (mean 2.9), reflecting circumstances such as being ‘released in new city’ (mean 2.2), ‘released on weekends (mean 2.9) and ‘released at an unscheduled time from jail or court’ (mean 2.8), and ‘Consequences of Imprisonment’ (mean 3.3), including statements such as ‘post-traumatic stress from prison experience’ (mean 3.1) and ‘disclosure of being in jail’ (mean 3.1).

**Table 2:** Statement Ratings

Domain	Statement number	Variable	Importance Rating	Feasibility Rating
Medical	1	Distrust of medical providers	3.61	3.44
	2	Uncaring attitude of medical staff	4.22	3.33
	4	Lack of medical records	3.94	3.72
	27	Untreated depression and mental health illness	4.33	3.67
Stigma	6	No self-esteem	3.72	3.61
	22	Substance use and addiction	4.72	3.28
	35	Fear of HIV disclosure	3.67	3.28
Consequences of imprisonment	15	Embarrassed about being imprisoned	3.06	2.83
	24	Disclosure of being in jail	3.06	3.06
	25	Post-traumatic stress from prison experience	3.94	3.11
	31	No communication between prison and workers or physician	4.22	3.83
	34	Do not know how to re-connect with providers	4.06	3.78
Prison release	8	Released at night	3.28	2.82
	9	Released on weekends	3.44	2.94
	1-	Unscheduled release from jail or court	3.72	2.78
	21	No pre-release preparation	4.22	3.17
	26	No information about services available upon release	4.17	3.83
	28	Isolated and alone on release	4.39	2.83
	30	Released from prison far from home	4.11	2.50
	33	Released in new city	3.89	2.22
Social connections	3	Establishing social support	4.61	4.22
	13	Regaining parental rights	4.00	3.44
	14	Afraid to draw attention to self because of fear of losing kids	3.61	3.06
	18	Reconnecting with family and children	4.50	3.89
	23	Reconnecting with ‘bad’ social network	3.44	3.28
Practical considerations	5	No transportation	4.17	3.44
	7	Need to meet conditions of parole	4.22	4.22
	12	Getting legal identification	4.44	4.28
	16	Re-instating disability and other income supports	4.67	4.17
	17	Re-instating drug coverage	4.72	4.33
Survival needs	11	Getting a job	4.56	3.39
	19	Safety and protection from abuse	4.50	3.28
	20	Securing housing	4.83	3.72
	29	Financial stress	4.44	2.61
	32	Need to secure food	4.72	3.61

**Fig. 2:**
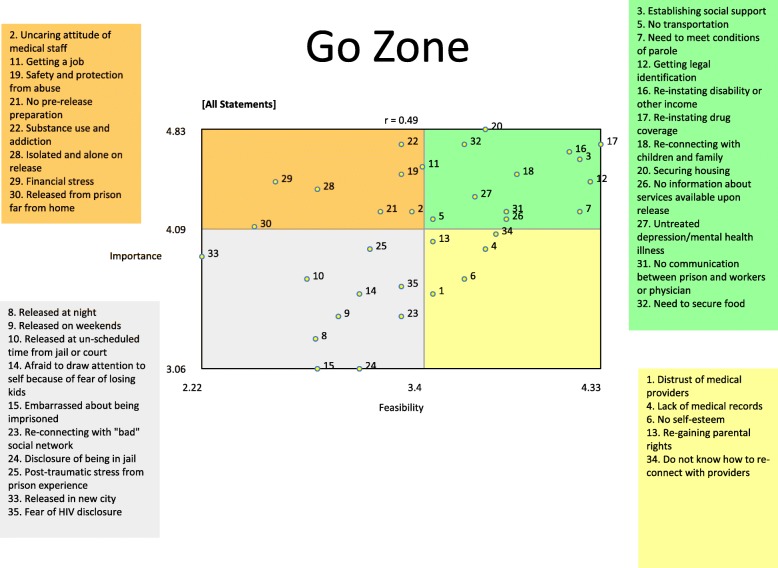
Go-zone analysis of importance and feasibility ratings

Importance and feasibility ratings were used to construct a ‘go-zone’ plot (Fig. 2), where items in the top right quadrant are those rated most important and feasible to address. Twelve of the 35 brainstormed statements located within six of the seven clusters were found within the ‘go-zone’ (Fig. 2), including all statements within the ‘Practical Considerations’ domain. Other statements within the go-zone were ‘untreated depression/mental health illness’, ‘no communication between prison and workers or physician’, ‘no information about services available upon release’, ‘establishing social support’, ‘re-connecting with children and family’, ‘securing housing’ and ‘need to secure food’. The ‘Stigma’ cluster was the only one not represented in the go-zone. Conversely, ten statements from within four clusters were located in the bottom left quadrant, representing challenges that were rated least important and feasible to address, including challenges related to time and location of release (‘released at night’, ‘released on weekends’, ‘released at unscheduled time from jail or court’, ‘released in new city’), consequences of imprisonment (‘embarrassed about being imprisoned’, ‘disclosure of being in jail’, ‘post-traumatic stress from prison experience’), stigma (‘fear of HIV disclosure’) and social and family matters (‘afraid to draw attention to self because of fear losing kids’, ‘reconnecting with ‘bad’ social network’). The overall correlation between relative importance and feasibility of statements was modest (*r* = 0.49).

**Fig. 3:**
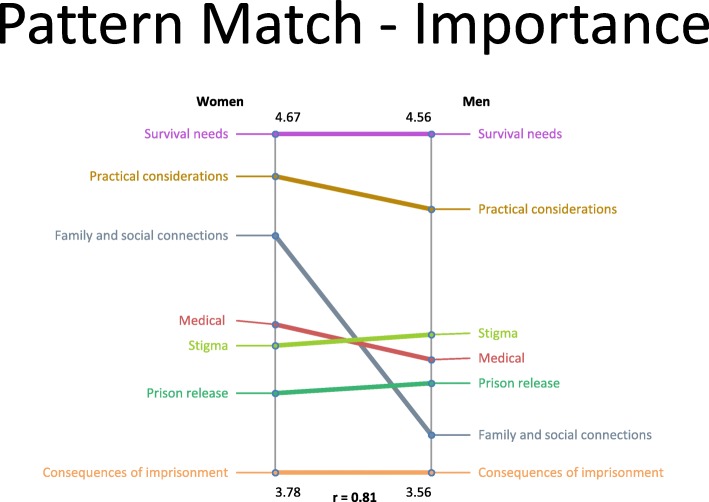
Pattern match comparison of cluster importance ratings between women and men

**Fig. 4:**
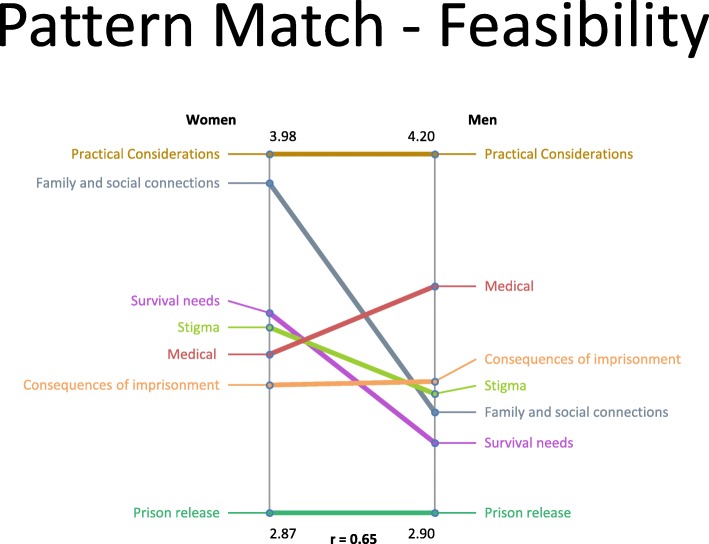
Pattern match comparison of cluster feasibility ratings between women and men

Pattern match graphs (Figs. 3 and 4) revealed some differences in importance and feasibility ratings between women and men. Although women and men rated ‘Survival Needs’ and ‘Practical Considerations’ the highest in terms of importance and feasibility, respectively, women assigned higher importance ratings to statements in the ‘Family and Social Connections’ cluster. In addition, women considered statements related to ‘Survival Needs’ more feasible to address than did men, and during discussion, stressed that limited access to resources encompassing the ‘Survival Needs’ cluster may lead some women to return to relationships in which they are at risk for ongoing abuse. Similarly, women highlighted the need for trauma informed services when developing post-release interventions. Women and men were similar in assigning relatively low importance and feasibility ratings for statements in the ‘Consequences of Imprisonment and ‘Prison Release’ clusters, respectively.

## Discussion

Using concept-mapping, we identified key challenges in accessing HIV-related care for formerly incarcerated people with HIV. Our findings underscore the important role of competing non-medical issues and logistical challenges at the time of release in delaying access to care and suggest that an ensuing reintegration program must successfully address these considerations.

Our study builds upon previous research examining barriers to re-connecting with care for formerly incarcerated people with HIV in several ways. First, we identified important differences between women and men in post-release priorities that may impact linkage with services. While findings from previous studies overlap with ours in demonstrating the importance of meeting subsistence needs [26], women in our study also highlighted the importance of reconnecting with family and children, such that mechanisms to support this process would be necessary components of future programming. In addition, the ensuing discussions about the important role played by abuse and trauma in the lives of formerly incarcerated women with HIV demonstrated the need for integrating trauma-informed services into transitional intervention programs. Although the provision of trauma-informed services in the context of HIV prevention has been well described, studies among individuals living with HIV are less common [27]. Furthermore, recent findings that 47.1% of a sample of Canadian women living with HIV have post-traumatic stress disorder related to past histories of abuse or violence reinforce the need for integrating trauma-informed services into HIV care more generally [28]. In addition, while most trauma-informed programs address trauma related to childhood abuse and intimate partner violence, post-traumatic stress related to incarceration and other forms of violence are noticeably absent from programs described in the literature [27]. Future research addressing these needs is required, as is exploring the role of providing trauma-informed services for formerly incarcerated men with HIV. Second, our study provides data on which challenges formerly incarcerated people with HIV perceive to be both the most important and feasible to address, insights which support subsequent knowledge translation by prioritizing areas for realistic intervention. The use of concept mapping permitted a visual transformation of these perspectives into easily interpretable maps representing the most salient obstacles to community re-entry. Because we were able to undertake these analyses immediately following the sorting and rating activities, participants were engaged with the visual maps in ‘real time’ and were able to provide concrete recommendations for the nature of services that would be required and specific partners to be engaged [29]. These participatory attributes of concept mapping distinguish our work from most prior studies in this field, where generated data are interpreted at a later time by the research team and may then be returned to the community for discussion, although this does not always occur. Finally, the finding that most clusters had high bridging values demonstrates that these constructs intersect with each other to manifest as barriers to post-release linkage, rather than operating in isolation. That is, because statements comprising these clusters were often sorted with those of other clusters, and therefore ‘bridge’ across domains, these statements may interact in specific ways or share common mechanisms for impeding post-release access to care. These findings suggest that a transitional intervention program would likely be most successful if it addressed multiple challenges simultaneously. The exception may be the ‘Prison Release’ cluster, where statements were commonly sorted together and may therefore reflect a more distinct concept requiring unique interventions.

Findings from this study informed subsequent stages in the development of a transitional intervention program for formerly incarcerated people with HIV. First, a peer researcher-initiated outreach to agencies that could address some of the principal challenges identified in the ‘go zone’, including food insecurity and housing. Individual meetings were arranged with each partner to gauge interest in supporting a transitional intervention program. Next, a peer-led half-day knowledge translation event was held with representatives of each agency, sharing findings of work to date and discussing how to operationalize a collaborative inter-agency partnership. Because of prior research suggesting low post-release linkage to care with transitional programs and that the inclusion of peer-navigators may augment the success of these interventions [30–32], it was decided that peer navigation would be a component of this program. A recently published randomized trial demonstrating greater virologic suppression at 12 months among formerly incarcerated individuals with HIV randomized to peer navigation relative to transitional case management alone (49.6% vs. 36.0%) reinforced the importance of integrating peers into the program [33]. Subsequent steps will be formalizing implementation and evaluation of the program.

Some limitations of our work merit discussion. First, participants were known clients of various organizations across Ontario who were recruited by staff of participating agencies, and may not be representative of the population of formerly incarcerated people with HIV. Our findings therefore may not be transferable to formerly incarcerated individuals with HIV who are less engaged with health and social services. However, we were interested in purposively sampling individuals who could speak to the experience of accessing care following incarceration, rather than probabilistic generalizability to the entire population of formerly incarcerated people with HIV in Ontario. Similarly, we did not study youth and were unable to successfully recruit members of the transgender community for our study. It is conceivable that members of these communities could experience challenges re-connecting with care following prison release differently from our study participants. We therefore invited agencies that provide services for youth and transgender individuals to collaborate on the implementation and evaluation of the intervention. Although our small sample size precludes generalizability to the larger population of formerly incarcerated people with HIV, this was not the intent of our study, and we had a sufficient number of participants for concept mapping methodology. We did not capture information about medication adherence, length of incarceration and time since release, precluding us from identifying response patterns associated with these variables. Finally, the application of our findings to other jurisdictions may be limited because perceptions of barriers are likely influenced by local context. However, the methods used in on our study can be applied in other settings to inform the development of programming for formerly incarcerated people with HIV.

## Conclusions

We used concept mapping to identify the main challenges faced by formerly incarcerated people with HIV attempting to engage with care following prison release and to further prioritize these barriers into those that would be most important and feasible to address with a transitional intervention program. Involvement of participants in the analysis and interpretation of data informed the development of an intervention by highlighting the primacy of meeting basic needs at the time of release, overcoming logistical barriers related to community re-entry and identifying agencies with which partnerships should be sought. Our work demonstrates how participatory methods such as concept mapping can facilitate the translation of research into practice, including for vulnerable populations such as formerly incarcerated people with HIV.

## Data Availability

All data generated or analyzed during this study are included in this published article.
